# Metabolic syndrome among adults aged 18–40 years in the United Arab Emirates: prevalence and associated factors using harmonized criteria

**DOI:** 10.3389/fpubh.2026.1868692

**Published:** 2026-09-23

**Authors:** Shamma AlMuhairi, Aminu S. Abdullahi, Fatma Al-Maskari, Luai A. Ahmed, Raghib Ali, Javaid Nauman, Syed M. Shah

**Affiliations:** 1 Institute of Public Health, College of Medicine and Health Sciences, United Arab Emirates University, Al-Ain, United Arab Emirates; 2 Tawam Hospital, Abu Dhabi Health Services Company (SEHA), Al Ain, United Arab Emirates, Al-Ain, United Arab Emirates; 3 Zayed Center for Health Sciences, United Arab Emirates University, Al-Ain, United Arab Emirates; 4 Public Health Research Center, New York University Abu Dhabi, Abu Dhabi, United Arab Emirates; 5 Department of Circulation and Medical Imaging, Faculty of Medicine and Health Sciences, Norwegian University of Science and Technology, Trondheim, Norway

**Keywords:** metabolic syndrome, prevalence, young adults, physical activity, harmonized criteria, United Arab Emirates

## Abstract

**Background:**

Metabolic syndrome (MetS) refers to a constellation of cardiometabolic risk factors that increase the risk of cardiovascular diseases and type 2 diabetes (T2DM). This study estimated the prevalence of MetS and its associated factors in young adults in the United Arab Emirates (UAE).

**Methods:**

This was a cross-sectional study. We collected data from participants aged 18–40 years in the UAE Healthy Future Study (*N* = 5,050). Demographic and health information was collected through self-reported questionnaires. Anthropometric data and blood pressure measurements were taken, and blood samples were collected for subsequent biochemical analyses.

**Results:**

According to the harmonized criteria, the overall prevalence of MetS was 22.4% (95% confidence interval: 21.2–23.5%). MetS was more prevalent in men (26.1%) than in women (16.7%). Elevated blood pressure (40%) was the most prevalent component of MetS, followed by abdominal obesity (39%) and reduced HDL-c (37%). Approximately 7% of the participants had all five components of MetS. Significant risk factors for MetS in the studied cohort included male sex, older age, being married, and a lower level of education.

**Conclusion:**

MetS was highly prevalent in our young study population, affecting approximately one in four young Emiratis. Population-based control strategies that focus on a healthy lifestyle, including physical activity and a healthy diet, are strongly recommended to decrease the future burden of T2DM and cardiovascular diseases.

## Introduction

Noncommunicable diseases (NCDs) contribute to an estimated 70% of premature mortality in the United Arab Emirates (UAE) (1). Obesity is rapidly increasing among adults in the UAE, and increased obesity reportedly leads to dyslipidemia, T2DM, hypertension, and other metabolic abnormalities, resulting in an increased risk of atherosclerotic cardiovascular disease (CVD) (2).

In countries with a high incidence of T2DM, such as the Pacific and the Middle East, including the UAE, one in four deaths in adults aged 35–64 years is caused by CVDs (3). Metabolic syndrome (MetS) is characterized by a cluster of cardiovascular risk factors, including abdominal obesity, elevated blood pressure, impaired glucose concentration, and dyslipidemia (4). This syndrome is associated with the development of T2DM, CVD, and an increased risk of all-cause mortality (5). A recent meta-analysis by Motillo et al. indicated that MetS doubles the risk of CVD outcomes and increases all-cause mortality by 1.5 times (6). MetS has a significant impact on public health owing to its progressively increasing prevalence in different ethnic groups at different ages (7). The increasing prevalence of obesity in the UAE is associated with an increased prevalence of MetS among college students, adolescents, and elementary school children aged 6–11 years (8–10).

Previous studies documenting MetS in Emirati adults used the International Diabetes Federation (IDF) (3) and Adult Treatment Panel III (ATP III) criteria (11), including the first study published in 2008 on the prevalence of MetS in the multiethnic population of the UAE, based on a national survey report, which revealed that the age-adjusted prevalence of MetS using the ATP III definition was 39.6% (12). The second study conducted in 2008 exhibited that the prevalence of the MetS based on the modified ATP III criteria was 25.2% (13). The third study, conducted in 2008, using the IDF and ATP III criteria, revealed that the prevalence of MetS was 39.6 and 40.5%, respectively (14). The most recent study documenting the prevalence of MetS, conducted in the Sharjah Emirate of the UAE, also used the ATP criteria to define MetS and demonstrated that the overall prevalence was 37.4% (15). However, none of the UAE studies have used the most recent definition of MetS, which is the consensus definition that incorporates all previous definitions and is called the harmonized definition or Joint Interim Statement (JIS) of MetS (16). The use of different criteria by major organizations to define MetS remains a concern when comparing the burden of MetS across countries and populations. By consolidating the core components of ATP III and IDF into a single consensus framework, the Joint Interim Statement (harmonized) definition minimizes prevalence differences driven purely by definitional variation and thereby strengthens epidemiological comparability across studies and populations. This harmonization resolves key discrepancies between earlier criteria, particularly around central obesity requirements and threshold conventions, allowing more consistent cross-study benchmarking and clearer interpretation. The primary objective of this study was to estimate the prevalence of MetS, defined according to the harmonized criteria, in a large sample of young Emirati adults aged 18–40 years. The secondary objective was to identify factors associated with MetS using the same criteria.

## Methods

### Study design and sampling

We collected data from the UAE Healthy Future Cohort Study (UAEHFS) to identify the causes of chronic diseases, including diabetes and CVDs (17). The UAEHFS is a population-based prospective cohort study that recruited 20,000 adult Emiratis aged 18–40 years between February 2016 and December 2018. Participation in the study was purely voluntary, and recruitment was conducted through primary health care centers or satellite clinics established by the research team. Further details are described previously (18).

### Data collection

The participants (*N* = 5,050) answered a self-completed questionnaire that collected socio-demographic, health, and lifestyle information and underwent physical examinations, including measurements of height, weight, waist and hip circumferences, and blood pressure. Fasting blood samples were collected to measure glycated hemoglobin, low-density lipoprotein (LDL-c), high-density lipoprotein (HDL-c), total cholesterol, triglyceride levels, and blood glucose levels.

### Definitions

Body mass index (BMI) was categorized according to the WHO definition: 18.5–24.9 kg/m^2^ was considered normal weight, 25.0–29.9 kg/m^2^ overweight, and ≥30.0 kg/m^2^ obesity.

The consensus definition of MetS (the harmonized definition) states the fulfilment of any three of the following criteria: elevated waist circumference (according to population and country-specific definitions, so IDF middle East/North Africa value is ≥94 cm for men and ≥80 cm for women) (3), triglyceride level ≥150 mg/dL, HDL-c level <40 mg/dL in men and <50 mg/dL in women, blood pressure ≥130/85 mmHg, or fasting glucose level ≥100 mg/dL (16).

### Ethical considerations

Informed consent was obtained from all the participants. Participants who reported any acute infection at the time of recruitment or who were pregnant were excluded from the study. This study was approved by the Abu Dhabi Health Research and Technology Committee, the Institutional Review Board at New York University Abu Dhabi, the Ministry of Health and Prevention in the UAE, and the Dubai Health Authority (ref. DOH/HQD/2020/516) (17).

### Data analysis

Continuous and categorical variables are presented as means (standard deviations) and frequencies (percentages), respectively. Proportions and corresponding 95% confidence interval estimates were reported for MetS and its components. To facilitate comparisons with rates from other studies, age-standardized prevalence rates were calculated for MetS. Age-standardized prevalence was estimated using the direct standardization method, based on the WHO World Standard Population (2000–2025). Age-specific prevalence estimates were weighted according to the corresponding WHO standard population distribution for the age groups represented in the study. Differences in the prevalence of MetS within the categories of socio-demographic characteristics were reported and statistically tested using the chi-squared test. Given the relatively high prevalence of MetS in our study population, modified Poisson regression with robust standard errors was employed to estimate prevalence ratios and identify factors associated with MetS. For the regression analyses, age was categorized as 18–24, 25–29, and 30–40 years. Marital status was categorized as married or unmarried (single, divorced, separated, or widowed), while education was categorized as tertiary education (undergraduate or postgraduate) or high school education or lower to provide epidemiologically interpretable comparisons and avoid small categories. Missing data were handled using complete-case analysis, whereby participants with missing data for any covariate included in the multivariable model were excluded from the adjusted analysis. Statistical significance was set at *p* < 0.05. Data analysis was performed using R version 4.4.1.

## Results

A total of 5,050 participants were included in this cross-sectional analysis of the cohort data, with the majority (60%) being male (Table 1). The mean age of the participants was 26 years (SD = 6.2), with the majority (52.4%) aged 18–24 years. Most participants were single (63.6%). Furthermore, 53, 40.5%, and approximately 6% of the study population held high school certificates, undergraduate degrees, and postgraduate degrees, respectively. Approximately four in five participants had a monthly family income of at least 20,000 AED (≈5,440 USD). Approximately 27% were current smokers. The participants had a mean systolic and diastolic blood pressure of 125.5 mmHg (SD = 14.1) and 77.8 mmHg (SD = 10.1), respectively. The average waist and hip circumferences were 86.2 cm (SD = 15.8) and 104 cm (SD = 12.7), respectively. The average fasting blood glucose level was 96.1 mg/dL (SD = 28.5). The mean LDL-c, HDL-c, and triglyceride levels were estimated at 115.8 mg/dL (SD = 34.0), 48.5 mg/dL (SD = 13.0), and 103.9 mg/dL (SD = 82.8), respectively. Overall, the participants with and without MetS differed significantly in terms of age (*p* < 0.001), sex (*p* < 0.001), marital status (*p* < 0.001), education level (*p* = 0.016), and smoking status (*p* = 0.006).

**Table 1:** Prevalence of metabolic syndrome using the JIS criteria, stratified by characteristics of participants (*n* = 5,050).

Characteristic	All participants	Metabolic syndrome		*p*-value
	*N* (%)	*n*	Prevalence and 95% CI	
Age				<0.001
18–24	2,645 (52.4)	389	14.7 (13.4–16.1)	
25–29	1,024 (20.3)	243	23.7 (21.2–26.4)	
30–34	760 (15.0)	261	34.3 (31.0–37.8)	
35–39	572 (11.3)	216	37.8 (33.9–41.8)	
40	49 (1.0)	21	42.9 (29.6–57.3)	
Sex				<0.001
Male	3,044 (60.3)	795	26.1 (24.6–27.7)	
Female	2,006 (39.7)	335	16.7 (15.1–18.4)	
Marital status				<0.001
Single	2,876 (63.6)	464	16.1 (14.8–17.5)	
Married	1,534 (33.9)	506	33.0 (30.7–35.4)	
Divorced/Separated/Widowed	114 (2.5)	31	27.2 (19.8–36.2)	
Level of education				0.016
High school or lower	2,362 (53.4)	550	23.3 (21.6–25.0)	
Undergraduate	1,792 (40.5)	357	19.9 (18.1–21.8)	
Postgraduate	271 (6.1)	68	25.1 (20.3–30.6)	
Monthly income (AED)				0.653
<20,000	552 (21.0)	131	23.7 (20.4–27.5)	
20,000–39,999	976 (37.1)	235	24.1 (21.5–26.9)	
40,000–59,999	418 (15.9)	99	23.7 (19.8–28.0)	
> = 60,000	683 (26.0)	147	21.5 (18.6–24.8)	
Smoking				0.006
No	3,679 (72.9)	787	21.4 (20.1–22.7)	
Yes	1,371 (27.1)	343	25.0 (22.8–27.4)	

CI: Confidence interval; SD: Standard deviation. Totals may not sum to 5,050 because of missing data.

The overall prevalence of MetS based on the harmonized criteria was estimated at 22.4% (95% confidence interval [CI]: 21.2–23.5%). The overall age-standardized prevalence of MetS was 29.2% (95% CI: 27.2–32.6). Table 2 presents the prevalence of the individual components of MetS according to the harmonized criteria, overall and stratified by sex. Elevated blood pressure was the most prevalent component (40%), followed by abdominal obesity (39%) and reduced HDL-c (37%). The prevalence of most MetS components increased with age in both women and men, with abdominal obesity showing the most pronounced increase with age, particularly among women (Figure 1). Men had significantly higher prevalences of elevated blood glucose, elevated triglycerides, and reduced HDL-c than women, whereas the prevalences of abdominal obesity and elevated blood pressure did not differ significantly between sexes (Table 2). Moreover, among participants with MetS, the majority (63%) had only the minimum number of MetS factors, i.e., three; 31% had four, whereas only 7% had all five factors. The prevalence of MetS increased significantly with increasing BMI (*p* < 0.001) in both men and women (Figure 2).

**Table 2:** Prevalence of the individual components of metabolic syndrome according to the harmonized (JIS) criteria, overall and stratified by sex.

Characteristics	Total (*N* = 5,050)		Men (*n* = 3,044; 60.3%)		Women (*n* = 2,006; 39.7%)		*p*-value
	*n*	%	*n*	%	*n*	%	
Abdominal obesity	1,982	39%	1,203	40%	779	39%	0.600
Elevated blood pressure	2,008	40%	1,192	39%	816	41%	0.300
Elevated blood glucose	1,319	26%	952	31%	367	18%	<0.001
Elevated triglycerides	921	18%	747	25%	174	9%	<0.001
Reduced HDL cholesterol	1,835	37%	1,145	38%	690	35%	0.022

HDL, high density lipoprotein.

**Figure 1:**
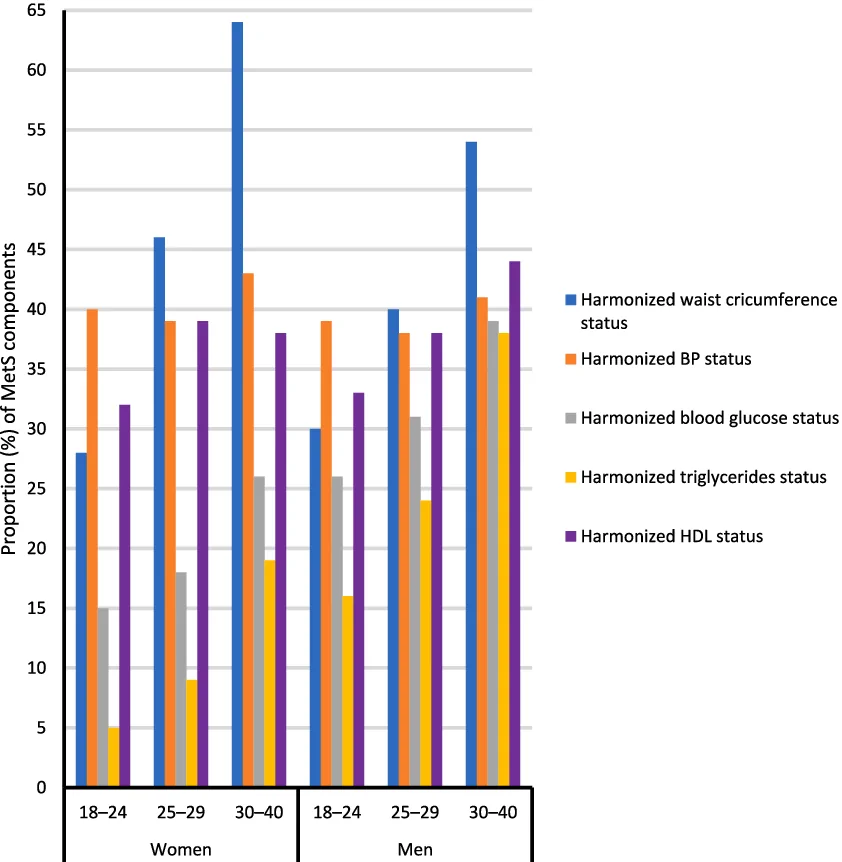
Prevalence of metabolic syndrome components according to age group, stratified by sex.

**Figure 2:**
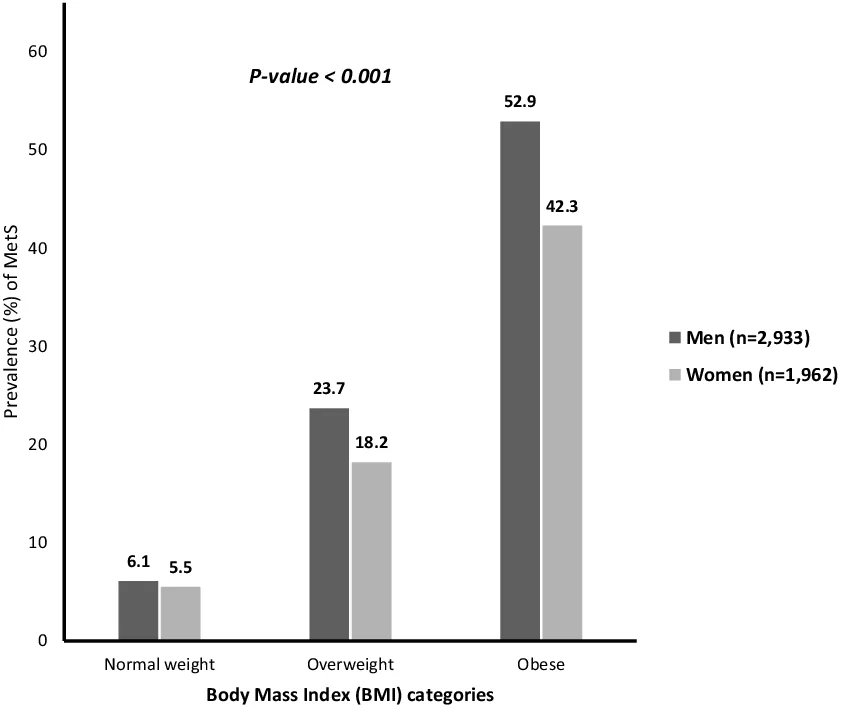
Prevalence of metabolic syndrome according to body mass index (BMI) categories among participants with available BMI data (*n* = 4,895), stratified by sex.

The prevalence of MetS was significantly different (*p* < 0.001) between men and women, with men showing a higher prevalence (26.1% vs. 16.7%) (Table 1). Age was also significantly associated with MetS (*p* < 0.001), with the highest prevalence among those aged 40 years (42.9%), followed by those aged 30–39 years (35.8%) and those aged 18–29 years (17.2%). Similarly, the prevalence of MetS was significantly higher among married participants (*p* < 0.001) at 33% compared to those in other marital status categories (16–27%). Regarding the level of education, MetS was significantly (*p* = 0.016) more prevalent among postgraduate degree holders (25.1%) than among undergraduate degree holders (19.9%) or those with high school/lower grades (23.3%). The prevalence of MetS was significantly higher among smokers compared to non-smokers (25% vs. 21.4%, *p* = 0.006). Family income (*p* = 0.653) and depression symptoms (*p* = 0.664) were not significantly associated with MetS.

In the adjusted analysis, older age, male sex, marital status, and lower education were independently associated with MetS (Table 3). Compared with participants aged 18–24 years, those aged 25–29 years had a 58% higher prevalence of MetS (aPR = 1.58, 95% CI: 1.30–1.91), while participants aged 30–40 years had more than twice the prevalence (aPR = 2.18, 95% CI: 1.80–2.64). Males were more likely to have MetS than females (aPR = 1.36, 95% CI: 1.17–1.57), and married individuals had a higher prevalence compared with unmarried individuals (aPR = 1.24, 95% CI: 1.05–1.46). Participants with high school education or lower also had a higher prevalence compared with those with tertiary education (aPR = 1.36, 95% CI: 1.19–1.55).

**Table 3:** Univariable and multivariable modified Poisson regression analysis of factors associated with metabolic syndrome assessed using the harmonized criteria.

Predictor	cPR (95% CI)	*p*-value	aPR (95% CI)	*p*-value
Age				
18–24	1		1	
25–29	1.61 (1.37–1.89)	<0.001	1.58 (1.30–1.91)	<0.001
30–40	2.45 (2.15–2.80)	<0.001	2.18 (1.80–2.64)	<0.001
Sex				
Female	1		1	
Male	1.56 (1.38–1.78)	<0.001	1.36 (1.17–1.57)	<0.001
Marital status				
Unmarried	1		1	
Married	1.99 (1.76–2.26)	<0.001	1.24 (1.05–1.46)	0.013
Level of education				
Tertiary (undergraduate and postgraduate)	1		1	
High school or lower	1.13 (1.00–1.28)	0.058	1.36 (1.19–1.55)	<0.001
Smoking				
No	1		1	
Yes	1.17 (1.03–1.33)	0.016	1.01 (0.88–1.16)	0.900

cPR: crude prevalence ratio; aPR: adjusted prevalence ratio; CI: confidence interval. The multivariable analysis was based on 4,404 participants with complete data for all variables included in the model.

## Discussion

To our knowledge, this study is the first to use the harmonized criteria to estimate the prevalence of MetS in the UAE. Overall, the prevalence of MetS was 22.4%. The UAE has one of the highest prevalence rates of T2DM and obesity worldwide (12). Moreover, we found a high prevalence of MetS and each of its components. Most previous studies used either the ATP III or IDF criteria, with prevalence rates ranging from 14 to 39% (12–14, 20). This emphasizes the importance of using a unified criterion to obtain harmonized estimates. Gulf countries have experienced similar circumstances to the UAE community; hence, we expect similar prevalence ranges among them. However, nearly all studies used the ATP III or IDF criteria (21), except for a study by Roshdy, who examined the prevalence of MetS among patients with psychiatric disorders using the harmonized criteria and reported a prevalence of 27% (22). The prevalence of MetS in Canada, according to the harmonized criteria, was 19.1%, similar to our estimate, as both countries share comparable lifestyle characteristics, including a sedentary lifestyle and increasing fast food marketing (23).

Our study revealed that elevated blood pressure was the most prevalent component of MetS in our study (40%), followed closely by abdominal obesity (39%) and reduced HDL-c (37%). The prevalence of abdominal obesity was comparable to that reported in a Canadian study (35.0%) (23). This result was expected because the prevalence rates of obesity and overweight are very high in the UAE (12). In our study, MetS affected men more frequently than women, which is supported by numerous studies in Saudi Arabia, Jordan, and Canada (24). This finding was similar for all cardiometabolic risk factors, which were more predominant among men (15).

Parallel to the findings reported in many studies, including the American (25), Saudi (26), and Canadian studies (23), the prevalence of MetS increased with increasing age, which can be explained by the increasing prevalence of chronic diseases with age, including T2DM, hypertension, and dyslipidemia (27).

Another important factor associated with MetS was the educational level and marital status of the participants, supported by multiple other studies on cardiometabolic factors; people with lower education levels and those who are married tend to have bad habits, including smoking, reduced engagement with physical activity, and poor dietary habits (28).

In our study, the prevalence of MetS significantly increased with an increase in BMI (*p* < 0.001) in both men and women, which clearly indicates that obesity requires intensive treatment and is associated with an increased risk of MetS (29). The association between BMI and increased risk of cardiometabolic diseases, such as T2DM, is more than a mere dose–response relationship (30). Obesity is reportedly often accompanied by MetS (11). Its prevalence increases with increasing BMI (31, 32).

The prospects for clinical and assistive practice in MetS are increasingly shaped by the recognition that multifactorial, sustained lifestyle interventions are the cornerstone of both prevention and management. Recent randomized trials and meta-analyses demonstrate that habit-based lifestyle programs, combining dietary modification, physical activity, and behavioral support, can achieve sustained remission in a significant proportion of patients, though long-term maintenance remains challenging and requires ongoing engagement (33–37). The American College of Cardiology and American Heart Association recommend lifestyle therapies as first-line treatment, emphasizing their role in reducing atherosclerotic cardiovascular disease (ASCVD) and diabetes risk (38).

Assistive practice now incorporates structured, supervised interventions, digital health tools, and multidisciplinary care teams to optimize adherence and address barriers to care, particularly in high-risk and underserved populations (36, 37). The Endocrine Society highlights the utility of MetS as a risk-enhancing factor for targeted screening and individualized management, advocating for comprehensive assessment of all risk components and integration of emerging pharmacotherapies when lifestyle modification alone is insufficient (39).

Population-level strategies, including ongoing surveillance, health education, and reduction of structural inequities, are critical to mitigating the rising prevalence and health disparities associated with MetS (35, 36). The evolving clinical paradigm prioritizes patient-centered, evidence-based approaches, with a focus on early identification, sustained behavioral change, and coordinated care to prevent progression to T2DM and cardiovascular disease (40). It will be worth studying and incorporating such practice in the UAE population and opening new opportunities for future research.

Our study has many strengths. To our knowledge, this is the first study to use the harmonized criteria of MetS. Furthermore, it was a nationwide representative study of Emiratis as it involved approximately 5,000 participants. In addition, the use of clinical and laboratory investigations increased the accuracy of the diagnoses.

### Limitations

This study has several limitations. First, its cross-sectional design precludes causal inference regarding associations between MetS and the examined correlates. Second, recruitment relied on convenience sampling, which may introduce selection bias and limit the generalizability of the findings to all young Emirati adults. Third, blood pressure and biochemical markers were assessed at a single time point; although standardized procedures were used, single measurements may be affected by short-term biological variability or measurement error. Fourth, several covariates (e.g., education, marital status, and smoking) were self-reported and are therefore subject to reporting and recall bias. Finally, the present analysis did not incorporate potentially important determinants such as physical activity, detailed dietary/nutritional parameters, or more granular ethnicity/ancestry measures, which may contribute to residual confounding. Future longitudinal analyses in representative samples, with repeated measurements and more comprehensive behavioral and sociodemographic characterization, are warranted to better delineate risk pathways and to inform targeted prevention strategies.

## Conclusion

In this cross-sectional analysis of young Emirati adults, approximately one in four met criteria for MetS, with higher risk among men and those who were older, married, or had lower educational attainment, and with abdominal obesity emerging as a predominant and actionable component. These findings underscore the need for a life-course, prevention-oriented public health response in the UAE, prioritizing early identification of cardiometabolic risk in young adulthood and scaling culturally tailored interventions that improve diet quality, increase physical activity, and reduce obesogenic exposures across schools, workplaces, and communities. Regionally, population-level strategies—routine surveillance using standardized metrics, targeted health literacy and behavior-change programs, and policy measures that address structural inequities shaping access to healthy food and opportunities for physical activity—are critical to curb projected future burdens of T2DM and cardiovascular disease. Importantly, application of the Joint Interim Statement (harmonized) criteria strengthens cardiometabolic surveillance by providing a unified operational definition that aligns key elements of both ATP III and IDF, reduces definitional heterogeneity, and enhances comparability of prevalence estimates across studies and populations; this, in turn, supports benchmarking, trend monitoring, and evaluation of prevention programs in the UAE and the wider MENA region. Collectively, our data provide an evidence base to inform early, coordinated, and patient-centered prevention pathways and to guide regional planning for cardiometabolic health.

## Data Availability

The data analyzed in this study are not publicly available due to ethical restrictions. Requests for access should be directed to the UAE Healthy Future Study research team.
